# A case report of cerebral venous sinus thrombosis presenting with rapidly progressive dementia

**DOI:** 10.3389/fmed.2022.985361

**Published:** 2022-08-25

**Authors:** Yaqiang Li, Mei Zhang, Min Xue, Ming Wei, Jiale He, Chunhui Dong

**Affiliations:** ^1^Department of Neurology, The First Affiliated Hospital of Anhui University of Science and Technology (First People’s Hospital of Huainan), Huainan, China; ^2^Department of Neurology, People’s Hospital of Lixin County, Bozhou, China; ^3^Department of Radiology, The First Affiliated Hospital of Anhui University of Science and Technology (First People’s Hospital of Huainan), Huainan, China; ^4^Department of Laboratory, The First Affiliated Hospital of Anhui University of Science and Technology (First People’s Hospital of Huainan), Huainan, China

**Keywords:** cerebral venous sinus thrombosis (CVST), dementia, rapidly progressive dementia, idiopathic intracranial hypertension (IIH), anticoagulant

## Abstract

**Background:**

Cerebral venous sinus thrombosis (CVST) is a rare but serious and treatable cause of neurologic symptoms. Due to the variable clinical presentation, CVST was often misdiagnosed. According to published case reports, common clinical manifestations of CVST include headache, focal neurological deficit, epilepsy, papilledema, etc. It is rare, nevertheless, to mention cases of rapidly progressive dementia (RPD).

**Case presentation:**

We reported a case of a 62-year-old retired male accountant, a Han Chinese from eastern China, who initially presented with slow response and memory decline. Until 2 months later, his memory declined and slow response deteriorated significantly, and he could not even complete simple tasks like brushing his teeth, washing his face, washing his feet, and dressing himself, and sometimes developed fecal incontinence. His neuropsychological test demonstrated severe cognitive decline. The cerebrospinal fluid (CSF) studies revealed markedly high opening pressure (260 mm of water), and coagulation tests indicated a mild elevation of D-Dimer of 1.19 mg/L. The magnetic resonance venography (MRV) showed thrombosis of the left transverse sinus, sigmoid sinus, and jugular venous bulb and was diagnosed as CVST. He switched from subcutaneous low molecular weight heparin (LMWH) and transitioned to oral anticoagulants at the time of discharge. The repeated CSF studies revealed normal opening pressure. After 5 days of anticoagulant treatment, his symptoms considerably improved, and a 1-month follow-up revealed that he had fully healed with no signs of recurrence.

**Conclusion:**

This case demonstrated the clinical heterogeneity of CVST, which should be taken into account for differential diagnosis of RPD. This case study also offered fresh data for the categorization of the clinical traits and the diagnosis of CVST.

## Introduction

Cerebral venous sinus thrombosis (CVST) is a rare but serious and treatable cause of neurologic symptoms. Whereas CVST accounts for fewer than 1% of all strokes, which primarily affects younger adults and children, and is characterized by clinical signs and symptoms such as headache, nausea and vomiting, optic papilledema, limb paralysis, and epilepsy (1–4). The incidence of diagnosis and misdiagnosis is significant due to the complicated and diverse clinical presentations of CVST, and it has been reported in the literature that the misdiagnosed rate can be as high as 50% (5). The relative rarity of this disease, combined with the variable and subacute presentation of symptoms, is thought to contribute to the delay in diagnosis (6). The clinical presentation of CVST is highly heterogeneous, with headache symptoms being the most prevalent (7). However, rapidly progressive dementia (RPD) is rare as the first symptom of CVST (8). Here, we described a patient presenting with slow response and memory decline as the first symptom and had been misdiagnosed. He was finally diagnosed as CVST based on cranial magnetic resonance imaging (MRI) combined with MR venography (MRV). This case report describes a rare clinical manifestation of CVST, which will contribute to a better understanding of the clinical characteristics of CVST for better diagnosis in the future.

## Case presenting

**FIGURE 1:**
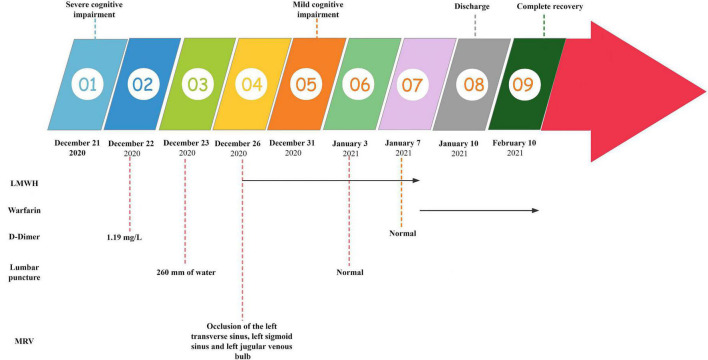
Summary of the clinical features, therapy, laboratory tests, and imaging tests. LMWH, low molecular weight heparin; MRV, magnetic resonance venography.

A 62-year-old retired male accountant, a Han Chinese from eastern China with a past medical history of significant hypertension and coronary artery disease, was admitted to a local hospital in September 2020 for “nephrotic syndrome” and was reported by his family to be cured and discharged on 50 mg of oral prednisone daily. The patient was admitted on December 21, 2020, for approximately 2 months for slow response and memory loss (Figure 1). He began to experience slow response, memory loss, personality change, irritability, and self-talk at times around October 2020, and the symptoms progressively worsened until 1 week ago, when he was unable to perform simple tasks like brushing teeth, washing face, washing feet, and dressing himself and sometimes had fecal incontinence. He denied complaints of fever, headache, slurred speech, numbness and weakness of the limbs, and convulsions. He visited a local hospital on December 12, 2020, and a head computed tomography (CT) indicated lacunar cerebral infarction with ischemic changes in the white matter of the brain. By using the Chinese medical treatment blood stasis-removing therapy using salvia miltiorrhiza, however, his symptoms deteriorated significantly.

On admission, he denied any history of smoking, alcohol abuse, or recreational drug use. He also had no known history of coronavirus disease-2019 (COVID-19) infection or vaccination with unremarkable family history. His body mass index at the time of admission was 25 kg/m^2^, with no change in weight over 1 year, vital signs were within normal limits, and his room air was well saturated. Neurological examination revealed slow response, memory loss, poor computation power (100–7 = 9, 9–7 = 0), and could only answer simple questions. His neuropsychological test revealed severe cognitive decline with a Mini-Mental State Examination (MMSE) score of 14 and a Montreal Cognitive Assessment (MoCA) score of 10. The muscle strength of his limbs was grade 5/5, the muscle tone, and tendon reflexes were normal, and the remaining physical and neurological examination results were not significant.

On day 2 after admission, relevant examinations were carried out. No abnormality was revealed by blood routine, routine urine and stool testing, thyroid function, glycosylated hemoglobin, and tumor markers, homocysteine. Some biochemical indications revealed mild abnormalities, such as total bilirubin of 34.00 μmol/L (normal range, 3.4–20.5 μmol/L), direct bilirubin of 11.00 μmol/L (normal range, 0–6.8 μmol/L), total protein of 52.3 g/L (normal range, 60–80 g/L), Albumin of 33 g/L (normal range, 34–48 g/L), Alanine aminotransferase of 56 U/L (normal range, 8–40 U/L), and lactate dehydrogenase of 340 U/L (normal range, 81–234 U/L). Coagulation tests also revealed mild elevation of D-Dimer was 1.19 mg/L (normal range, 0–0.55 mg/L). In addition, the hepatitis B surface antigen, syphilis antibody, hepatitis C virus antibody, and anti-human immunodeficiency virus antibody were negative. Lung CT plain scan showed bronchitis, coronary lesions, a small amount of pleural effusion on both sides, and thickened adhesions on the left pleura. Upper abdomen CT scan showed limited hepatic contour dysplasia and cholecystitis.

**FIGURE 2:**
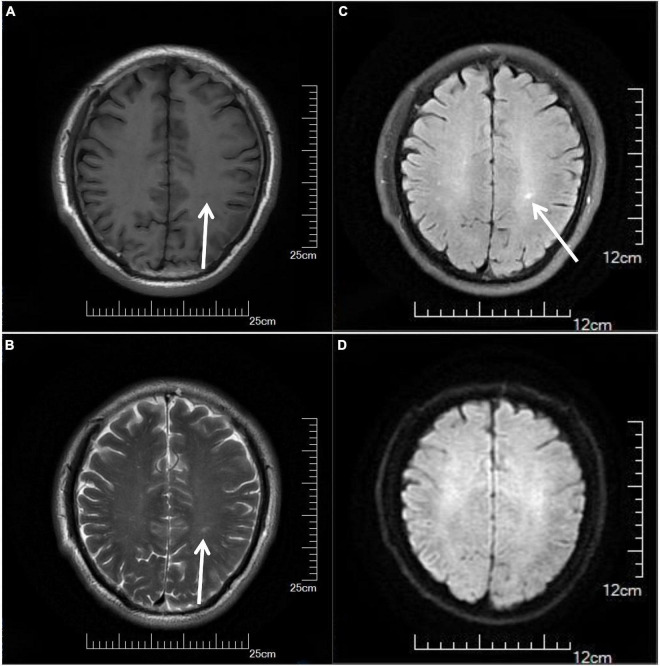
Cranial MRI results of the patient, showing multiple lesions in the center of the centrum semiovale with hypointensity on T1-weighted image **(A)** (arrows), hyperintensity on T2-weighted image **(B)** (arrows), on FLAIR **(C)** (arrows), and isointensity on DWI **(D)**. MRI, magnetic resonance imaging.

**FIGURE 3:**
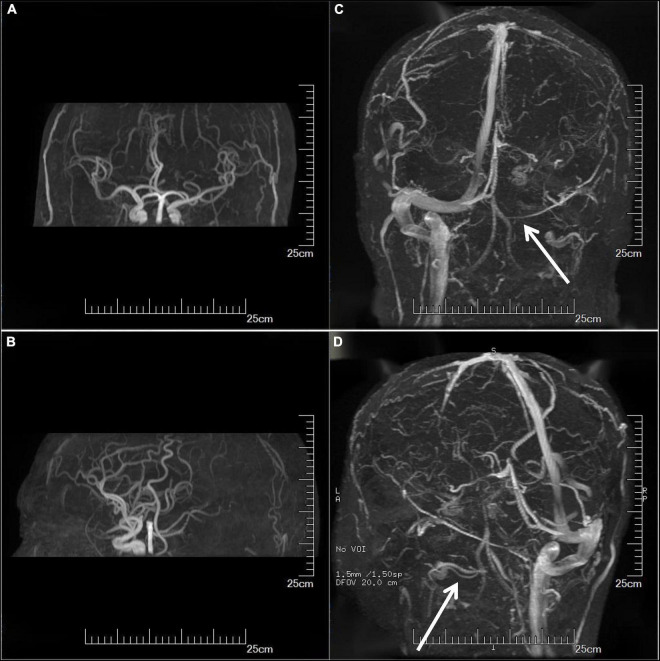
Imaging examination. Cranial MRA showed no significant intracranial aortic stenosis **(A,B)**. MRV showed thrombosis of the left transverse sinus, sigmoid sinus, and jugular venous bulb **(C,D)** (arrows). MRA, magnetic resonance angiography; MRV, magnetic resonance venography.

On the third day of admission, the lumbar puncture was performed, showing high opening pressure (260 mm of water), normal cerebrospinal fluid (CSF) protein level (276.5 mg/L), normal cell count (3 × 10^6/L), normal CSF glucose (4.2 mmol/L), and normal CSF chloride (125 mmol/L). Considering the probability of autoimmune encephalitis (AE), an antibody testing of CSF was also performed on December 25 using a cell-based assay (EUROIMMUN Medical Diagnostics). However, the anti-NMDAR, anti-AMPAR1, anti-AMPAR2, anti-mGluR5, anti-GAD65, anti-lgLON5, and anti-DPPX antibodies were detected as negative. Electroencephalogram (EEG) revealed extensive mild abnormal EEG (alpha generalization). Brain MRI exhibited multiple lesions in the center of the centrum semiovale, with hypointensity on T1-weighted image (Figure 2A), hyperintensity on T2-weighted image (Figure 2B), on FLAIR (Figure 2C), and isointensity on DWI (Figure 2D). No intracranial aortic stenosis was evident on cranial magnetic resonance angiography (MRA) (Figures 3A,B). On December 26, MRV demonstrated thrombosis of the left transverse sinus, sigmoid sinus, and jugular venous bulb (Figures 3C,D), which was considered CVST. Subsequently, the patients were screened for predisposing factors for thrombosis. Factor V Leiden and prothrombin gene mutations were negative. Furthermore, his protein S and protein C were all within normal limits. He was administered low molecular weight heparin (LMWH) 100 unit/kg subcutaneously twice daily to maintain APTT between 1.5 and 2.5 times of control value for 14 days. His symptoms improved significantly after 5 days of anticoagulation therapy, in which computing power was fully restored. The MMSE scores and MoCA scores were 22 and 18, respectively. A repeat lumbar puncture on January 3 showed normal opening pressures (160 mm water) and the rest of the CSF test results were within normal limits. The repeated coagulation tests also revealed a normal D-dimer on January 7. On day 3 prior to discharge, he transitioned to oral anticoagulants (warfarin) with anticipated treatment lasting 6 months. He was discharged on January 10 with complete resolution of his symptoms at the follow-up 1 month later. The MMSE scores and MoCA scores were 28 and 26, respectively with no sign of recurrence at the follow-up after 6 months. As for his cognition, the patient himself stated that there was no change in his memory and his family did not notice any change in his personality or behavior.

## Discussion

Goyal and colleagues summarized the clinical characteristics of 181 cases of CVST, most of which presented with the first and main clinical manifestations of headache. Other clinical symptoms of CVST included seizures, altered sensorium, focal neurological deficit, and vertigo (9). In the aforementioned study, no cases of RPD were mentioned. Even if the first symptom of the patient are slow response and memory decline, its progression was rapid. Here, we reported an RPD case with slow response and memory decline as the first symptom.

RPD is a group of RPD syndromes in which the decline in cognitive impairment to dementia is less than 2 years, or even months or weeks, and is partially reversible and treatable (10). RPD can be roughly categorized as primary or secondary. Primary RPD occurs in Creutzfeldt-Jakob disease (CJD), rapidly progressive types of other neurodegenerative dementias, encephalitis, and other diseases that typically cause severe neuronal damage in a relatively short period of time. Secondary rapid disease progression can occur in predominantly slowly progressive CNS diseases, such as Alzheimer’s disease with cerebrovascular disease or Lewy body pathology. There are many secondary factors in RPD, and the rate and reversibility of RPD progression vary widely among factors. RPD caused by a viral infection, immune deficiency, toxicity, and metabolic diseases progresses fastest but is more effective in treatment (11). Although this patient demonstrated remarkably rapid progression, this patient had no clinical manifestations of ataxia, myoclonus, or other involuntary movements, no cranial MRI with high signal in the basal ganglia and thalamus, and no EEG showing triphasic waves. Therefore, the diagnosis of CJD was not supported in this patient. Additionally, the CSF was negative for AE and paraneoplastic markers, which could further exclude AE and paraneoplastic encephalitis.

The etiology of the elevated intracranial pressure can be divided into idiopathic and secondary. Idiopathic intracranial hypertension (IIH) is a clinical syndrome of unknown etiology that presents with headache, optic papilledema, and other intracranial pressure elevations as the main manifestations (12). IIH mostly occurs in obese women of childbearing age, excluding patients with parenchymal brain lesions and venous sinus system disease (13, 14). Approximately 70% of patients with idiopathic cranial hypertension have a visual impairment, and some patients may experience progressive vision loss or even blindness (15, 16). Secondary intracranial hypertension usually refers to disorders of clear etiology such as occupying lesions, thrombotic disease, meningeal lesions, etc. (12, 17). Although lumbar puncture showed high CSF pressure, the patient had no clinical manifestations such as headache, vomiting, optic papilledema, and decreased visual acuity. Therefore, the diagnosis of IIH was also not supported. Finally, we performed MRV, which revealed thrombosis of the left transverse sinus, sigmoid sinus, and jugular venous bulb, with a possible diagnosis of CVST. This example emphasizes the challenges in detecting CVST because the diagnosis of CVST may have been delayed or prevented due to the unusual clinical characteristics. Diagnosing CVST can be extremely difficult because of the differences in clinical presentations and imaging findings. When the results of the cranial MRI and head CT are normal, CVST cannot be completely ruled out, necessitating further MRV screening. The cranial MRI and MRV examinations are simple to operate and can compensate for each other’s shortcomings. Combined cranial MRI and MRV examinations have been used as an effective tool for the clinical diagnosis and prognosis of CVST (18).

The imbalance between venous thrombosis and fibrinolytic mechanisms is the main pathophysiological mechanism of CVST. Any condition that causes a hypercoagulable state of blood, abnormal venous blood flow, and inflammatory response of the venous wall may lead to CVST. The etiology of CVST is multifactorial and diverse, mainly including infectious and non-infectious factors (19). The infectious factors of CVST mainly include meningitis, mastoiditis, otitis, sinusitis, and skin and gum infections. The main non-infectious factors include hereditary prothrombotic conditions (e.g., protein S deficiency, protein C deficiency, and antithrombin III deficiency), antiphospholipid antibody syndrome (APLS), autoimmune diseases (e.g., systemic lupus erythematosus, behçet’s disease, and vasculitis), malignancies, nephrotic syndrome, hematological diseases, and oral contraceptives. Nevertheless, the exact cause of the disease is still unknown in about 30% of CVST patients (20). Recently, many cases of COVID-19 infection causing intracranial venous sinus thrombosis have been reported (21–23). Endothelial dysfunction occurs during COVID-2019 infection, leading to platelet adhesion, leukocyte aggregation, complement activation, and cytokine release, which results in microvascular thrombosis (24). Malignancy can cause the blood to be in a hypercoagulable state, which may be the cause of CVST. Since there was no weight loss, loss of appetite, anemia, or weakness, and routine examinations such as blood counts, biochemical markers, and tumor indicators came back negative, malignancy appeared improbable. We observed that this CVST patient had a mild elevation of D-dimer on day 5 after admission, which seemed to better corroborate the diagnosis of CVST. A meta-analysis indicated that D-dimer may be a useful diagnostic tool in the management of patients with suspected CVST (25). Therefore, if a patient presents CVST-related clinical manifestations, risk factors, and elevated D-dimer we need to be highly suspicious of CVST and should aggressively improve cranial imaging to clarify the diagnosis. However, a negative D-dimer does not completely exclude CVST, and further cranial imaging is warranted.

We also found that the patient had been diagnosed with nephrotic syndrome (NS) 1 month prior to the onset of the disease, and although the patient’s family had described the disease was now cured. Furthermore, the patient was also not admitted for clinical manifestations associated with nephrotic syndromes, such as absence of massive proteinuria, hypoproteinemia, edema, and hyperlipidemia. Currently, there have been increasing reports of CVST in NS patients during treatment, and clinicians should be fully aware of this phenomenon (26–29). Recent studies have shown that hypercoagulability is one of the main pathogenic mechanisms for the development of CVST in patients (30, 31), and similarly, recent findings have shown that the blood of NS patients is in a hypercoagulable state (32). It is therefore hypothesized that the hypercoagulable state of blood in NS patients may be related to the occurrence of CVST. All reported risk factors for the hypercoagulable state in NS patients were summarized to draw the following conclusions (33, 34): (1) Hyperlipidemia: Increased synthesis of hepatic cholesterol, triglycerides, and lipoproteins in NS patients leads to hyperlipidemia and increased blood viscosity; (2) Hypoproteinemia: Decreased serum albumin results in systemic edema, fluid retention in the interstitial space, and insufficient effective blood volume, which leads to an increase in blood viscosity. In addition, because of the loss of protein, the liver compensates for the increase in protein synthesis, causing an imbalance in coagulation, anticoagulation, and fibrinolysis in the body; (3) Fibrinolytic system abnormalities: NS patients have a large amount of proteinuria, which reduces the protein content in the body and causes fibrinogen loss and hypoproteinemia, thus reducing the activity of the fibrinolytic system and further promoting a hypercoagulable state; (4) Increased fibrinogen: Higher fibrinogen can directly induce the aggregation of red blood cells and platelets, and increase blood viscosity, reducing blood flow, resulting in a hypercoagulable state of blood; (5) Thrombocytosis: NS patients have increased platelets and progressively reduced red blood cell deformability. In addition, the von Willebrand (vW) factor directs platelet aggregation to the vessel wall and increases platelet adhesion, and high platelet aggregation further aggravates the hypercoagulable state of the blood; (6) Iatrogenic factors: Patients with NS are hypovolemic due to massive diuretic use, which promotes thrombosis. Furthermore, NS patients receive long-term high-dose hormone therapy to treat primary disease, which can aggravate the hypercoagulable state of NS by stimulating platelet production, increasing the concentration of certain coagulation factors, aggravating the disorder of lipid metabolism, and decreasing fibrinolysis.

The patient’s symptoms improved significantly on day 5 of anticoagulation therapy with an MMSE score of 22 and a MoCA score of 18. Subsequently, the patients were prescribed oral anticoagulants (warfarin) to maintain INR between 2 and 3 for 6 months or longer depending on the underlying cause. The patient’s symptoms completely recovered 1 month after discharge, with an MMSE score of 28 and a MoCA score of 26. The treatment of CVST mainly consists of acute and long-term treatment, where the aim of the acute treatment is to prevent the progression of the thrombus and to recanalize the blocked sinus as much as possible, while the aim of the long-term treatment is to prevent the recurrence of the thrombus. Anticoagulation is the standard of care and the main therapy option for CVST patients throughout the acute period (35, 36). Anticoagulation therapy is effective in reducing the systemic hypercoagulability of CVST patients, reducing the spread and progression of thrombosis, and preventing the recurrence of CVST. Guidelines for CVST recommend that patients with CVST without contraindications to anticoagulation should be treated with anticoagulation as soon as possible, regardless of whether they have intracranial hemorrhage (36). Anticoagulants commonly administered to CVST patients in the acute phase included regular heparin and LMWH, with subcutaneous LMWH doses adjusted to patient weight being more effective and with a lower risk of bleeding than plain heparin (37). Patients with CVST should generally continue long-term treatment with oral anticoagulants, commonly warfarin, after the acute phase of anticoagulation; In principle, warfarin should be repeated with LMWH for 3–5 days, and LMWH should be discontinued after the maintenance of the INR between 2 and 3, and the warfarin dosage should be adjusted periodically to maintain the INR between 2 and 3 in accordance with the INR (5). However, the duration of oral anticoagulant therapy should be considered on the basis of individual genetic factors, triggers, recurrence, follow-up, and possible bleeding risk.

This patient had an excellent response to treatment. As a result, the patient’s extraordinarily good treatment success and full recovery from cognitive impairment constitute a significant strength of this study. Nevertheless, our study has several limitations. First, this is a case study of only one individual and therefore no broad conclusions or recommendations may be drawn. Second, although the patient’s MRV demonstrated thrombosis, the exact cause of the thrombosis was not found. Third, we did not conduct a second MRV examination to determine whether the high signal defect of blood flow in the implicated venous sinus was reevaluated.

In summary, the clinical heterogeneity of CVST increased the difficulty and complexity of our diagnosis. This case provides an excellent example of the utility of diagnostic imaging in the diagnosis of rare diseases such as CVST, which should be taken into account for differential diagnosis of RPD. This case study also provides new information for the diagnosis of CVST and the classification of the clinical characteristics.

## Data availability statement

The raw data supporting the conclusions of this article will be made available by the authors, without undue reservation.

## Ethics statement

Written informed consent was obtained from the individual for the publication of any potentially identifiable images or data included in this article.

## Author contributions

YL wrote the case report under the guidance of MZ. MX was the neurologist who treated the patient. MW from radiology was responsible for the interpretation of images. All authors were involved in making appropriate changes as needed and approved the final case report.
